# A systematic literature review of individuals’ perspectives on privacy and genetic information in the United States

**DOI:** 10.1371/journal.pone.0204417

**Published:** 2018-10-31

**Authors:** Ellen W. Clayton, Colin M. Halverson, Nila A. Sathe, Bradley A. Malin

**Affiliations:** 1 Center for Biomedical Ethics and Society, Vanderbilt University Medical Center, Nashville, TN, United States of America; 2 Department of Pediatrics, Vanderbilt University Medical Center, Nashville, TN, United States of America; 3 Center for Genetic Privacy & Identity in Community Settings, Vanderbilt University Medical Center, Nashville, TN, United States of America; 4 Vanderbilt Evidence-Based Practice Center, Institute for Medicine and Public Health, and Department of Health Policy, Vanderbilt University Medical Center, Nashville, TN, United States of America; 5 Departments of Biomedical Informatics and Biostatistics, Vanderbilt University Medical Center, Nashville, TN, United States of America; 6 Department of Electrical Engineering and Computer Science, Vanderbilt University, Nashville, TN, United States of America; Edith Cowan University, AUSTRALIA

## Abstract

Concerns about genetic privacy affect individuals’ willingness to accept genetic testing in clinical care and to participate in genomics research. To learn what is already known about these views, we conducted a systematic review, which ultimately analyzed 53 studies involving the perspectives of 47,974 participants on real or hypothetical privacy issues related to human genetic data. Bibliographic databases included MEDLINE, Web of Knowledge, and Sociological Abstracts. Three investigators independently screened studies against predetermined criteria and assessed risk of bias. The picture of genetic privacy that emerges from this systematic literature review is complex and riddled with gaps. When asked specifically “are you worried about genetic privacy,” the general public, patients, and professionals frequently said yes. In many cases, however, that question was posed poorly or only in the most general terms. While many participants expressed concern that genomic and medical information would be revealed to others, respondents frequently seemed to conflate privacy, confidentiality, control, and security. People varied widely in how much control they wanted over the use of data. They were more concerned about use by employers, insurers, and the government than they were about researchers and commercial entities. In addition, people are often willing to give up some privacy to obtain other goods. Importantly, little attention was paid to understanding the factors–sociocultural, relational, and media—that influence people’s opinions and decisions. Future investigations should explore in greater depth which concerns about genetic privacy are most salient to people and the social forces and contexts that influence those perceptions. It is also critical to identify the social practices that will make the collection and use of these data more trustworthy for participants as well as to identify the circumstances that lead people to set aside worries and decide to participate in research.

## Introduction

Genomics research often requires gathering data about genomic variation, phenotypes, demographics, and exposures from large numbers of people. Research using these data has the potential to uncover the contributions of genomic variants to human health and disease and provides a powerful tool for understanding how individuals’ life experiences affect outcomes. Discoveries can then be leveraged to develop more refined clinical care to improve individuals’ health, as already occurs in pharmacogenomics, [1–4] cancer diagnosis and therapy, [5] and solving previously undiagnosed diseases. [6–8]

In order to create sufficiently large collections of data for this type of research, investigators who plan to collect genomic, as well as other types of, data using funding from the U.S. National Institutes of Health (NIH) are now required—in almost all cases—to obtain express consent from participants for broad data sharing, which provides other investigators the freedom to use the data for a wide array of research endeavors without seeking additional consent. [9] As of 2017, the U.S. Common Rule, which regulates research with human subjects, now, for the first time, endorses broad data sharing as an acceptable option. [10] Despite significant public support for broad data sharing for research, [11] not all people in the United States are willing to allow genomic, and other related, data about themselves to be used for this or other purposes.[12, 13] One particularly prominent concern that people indicate is that uses of data about them for research impinges on their privacy. Privacy, however, is a multifaceted concept [14] that is often used by investigators and research subjects alike in a merely vaguely defined way–if they define it at all. Thus, it is necessary to parse various concepts encompassed by the expansive term of privacy to understand what underlies their apprehension.

The multiple connotations of privacy vary significantly in their implications for genomics research. One view of privacy is *solitude*, which is simply the desire to be “let alone.” [15] Privacy, at times, also refers to *anonymity*, or the wish not to be identified or otherwise made known to be a member of a group or class of persons. [16] A related concept that is frequently conflated with privacy is *confidentiality*, whereby a person can share information without making it widely available if the recipient, such as a primary care physician or psychotherapist, is legally or ethically obligated to keep secret what has been communicated to them by the sharer. [17] While privacy and confidentiality are analytically quite distinct, these terms are often used interchangeably in research settings [18, 19] and in casual conversation. This is a concern as the conflation has the potential to create confusion and a lack of clarity in characterizing individuals’ views about genetic privacy. These three notions–the desire to be let alone, to be anonymous, and to be protected by confidentiality–relate to how a person expects to be treated or regarded and so can be thought of as dignitary interests.

Dignitary concerns are not all that is at stake in discussions of genetic privacy. People also worry that genomic data about them will be used to ends with which they do not agree [20] or which might even harm them. In this respect, they are particularly concerned that data will be exploited to deny them, or those they love, access to important goods of life, such as employment and insurance. [21] As a result, people often seek to control how data about them are used and express worries about the efficacy of the affiliated governance and data security routines set in place. They do this even though examples of misuse and harm have been hard to come by, calling into question whether worries about widespread misuse and discrimination are well-founded. [22, 23] Nonetheless, concerns about potential harms have been a major topic of investigation and commentary. [24–26]

Understanding what people are worried about when they express their concerns in the phrase genetic privacy is an essential step toward developing strategies to take their concerns into account and, if possible, to allay them when developing organizational practices, setting policy in the United States, and designing privacy enhancing technologies. In order to learn what is already known and to identify additional empirical research questions whose answers could help inform policy development, we performed a systematic review of the qualitative and quantitative studies that have been conducted to date that have explored individuals’ views about the many aspects of genetic privacy in the context of clinical care and genetic research, as well as the demographic characteristics with which those concerns have been associated.

As will be illustrated in depth throughout this article, we discovered that research to date reveals a complex, but also incomplete, picture of what people think about genetic privacy. This is partially an artifact of the observation that the studies varied widely in what issues they explored. For instance, some studies asked simply whether people worried about genetic privacy, while others were more nuanced about the specific aspects of privacy they inquired about. The issues most frequently explored were opinions about how other people might use genetic information about an individual. In general, researchers were often thought to be trustworthy, especially if they were associated with the institution where the person sought care as a patient, while people worried about what employers, insurers, and the government would do with the data. Less attention was paid to the value of informed consent, access control, and governance, including the efficacy of legal protections. Respondents were rarely asked about re-identification risk (i.e., ascertaining the personal name or contact information of the individual to whom genetic data, devoid of identifiers, correspond) despite its prominence in policy debates in the United States and elsewhere, especially as compared with the greater frequency with which concerns about already identified data were probed. Of particular note, very little was asked about the social factors and relationships that often influence opinions. Still, despite the lack of detail about what people think about genetic privacy, a growing number of publications reveal that many people are willing to forgo some privacy to obtain genetic information and to promote research.

## Results

### Article selection and overview

**Fig 1:**
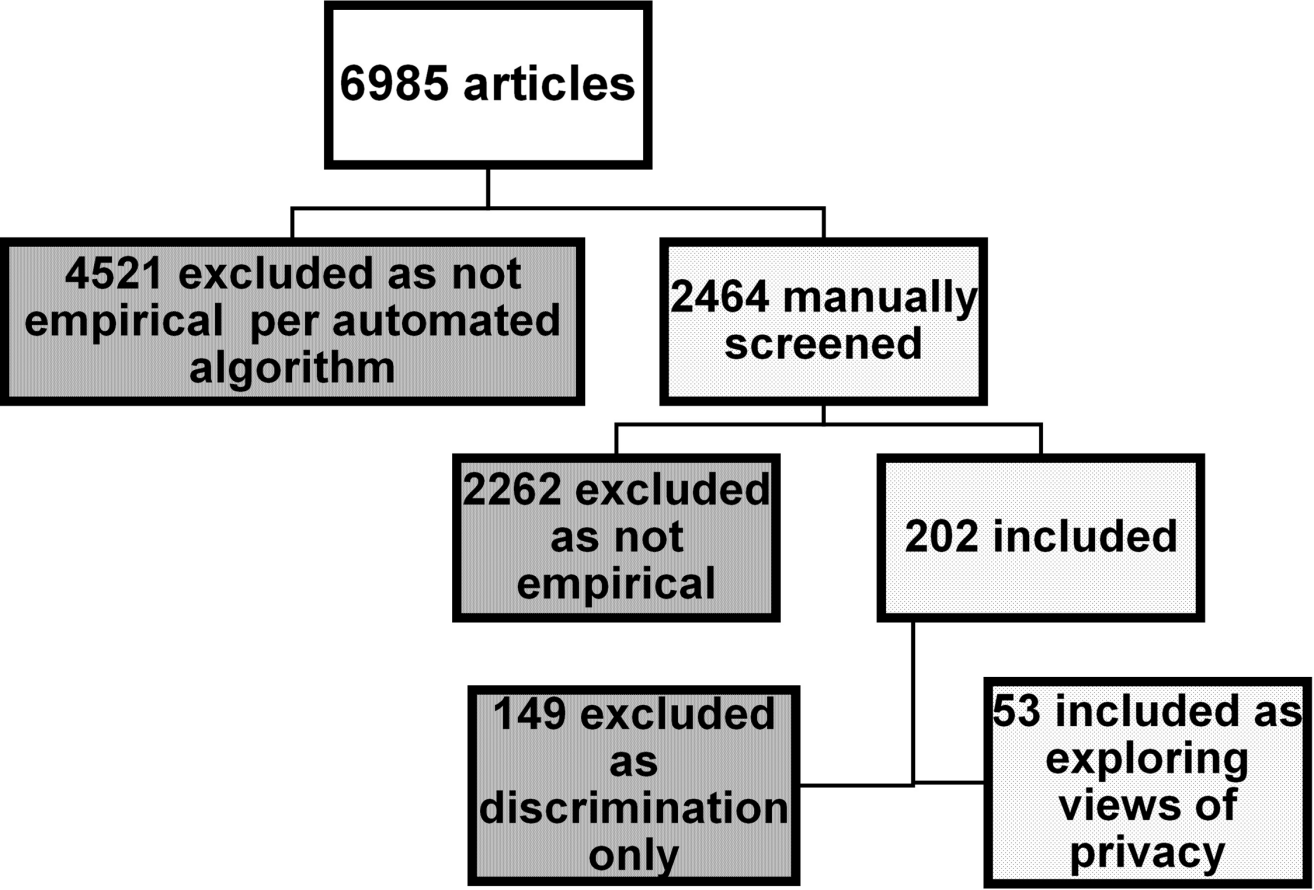
The process by which articles were selected and triaged during systematic review.

Our search retrieved 6,985 citations, 4,521 (64.7%) of which were triaged by the automated algorithm as not likely providing empirical data. Among the remaining 2,464 studies that were manually screened, 203 (8.2%) studies (reported across 217 publications) met our inclusion criteria and addressed aspects of privacy. However, the majority focused only on aspects of discrimination and so were excluded from further analysis. Fifty publications addressing broader perceptions of privacy as a major focus were supplemented by three additional articles following a manual search in July 2017, and it is these publications that are the subject of this review. (Fig 1 and S1 Table) Most studies (n = 36) were published between 2010 and 2017. We rated 4 studies as good, [12, 27–29] 35 studies as fair, [16, 18, 19, 28, 30–63] and 14 as poor quality. [62–76] (S2 Table) Studies with poor methodologic quality typically did not provide as much detail about personal views, but were retained to illustrate what has been explored in previous research.

The studies explored the responses of a total of 47,974 participants. Surveys including those in mixed methods studies were administered to 42,754 respondents, while a number of different qualitative approaches involved 11,327 participants. Eighteen studies, involving 35,323 respondents, explored the views of the general public and direct to consumer users. By contrast, 31 studies, involving 11,621 respondents, focused on patients, their families and surrogate decision makers, as well as research participants. The remainder of the studies reported on the views of clinicians, researchers, institutional review boards (IRBs), and journal editors, representing a different set of stakeholders. Observations from the latter studies are presented after those of the public, patients, and research participants.

Studies typically focused on specific contexts, such as genetic research generally (n = 13), [31, 32, 34, 46, 49, 60, 62, 63, 65, 74–77] biobanking or specimen donation (n = 17), [12, 16, 27–30, 39, 44, 45, 50, 51, 53, 54, 61, 68, 70, 71] with two studies reported in one publication.[28] Twelve studies focused on clinical genetics (including genetic testing and whole genome sequencing (WGS)), [19, 35–37, 42, 58, 59, 66, 67, 69, 72, 73] Most studies addressed views of the general public [35, 59, 72] or patients. [36, 37, 42, 69, 73] Three addressed direct-to-consumer (DTC) genetic testing. [18, 56, 67] Most addressed hypothetical questions about what people thought rather than decisions about genetic testing that people had experienced.

**Fig 2:**
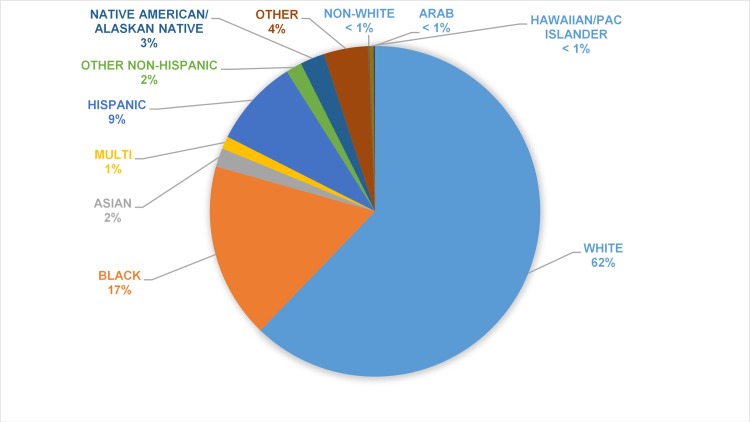
Race and/or ethnicity of the participants when they were identified in the study.

Of the studies reporting data regarding sex of the general public/patient participants (n = 41,519 participants), 26,656 (64%) were female. None addressed gender identity as distinct from reported sex. Across the 39 studies reporting data on race or ethnicity (n = 38,736 participants), 24,566 participants who completed participation identified themselves as White or Caucasian (63%), 6,430 as Black or African-American (17%), 3174 as Hispanic (8%),and 4511 as other (12%), a category which includes Asian and Native Americans and many other ethnic groups, as described in Fig 2.

### Issues explored in the studies

**Fig 3:**
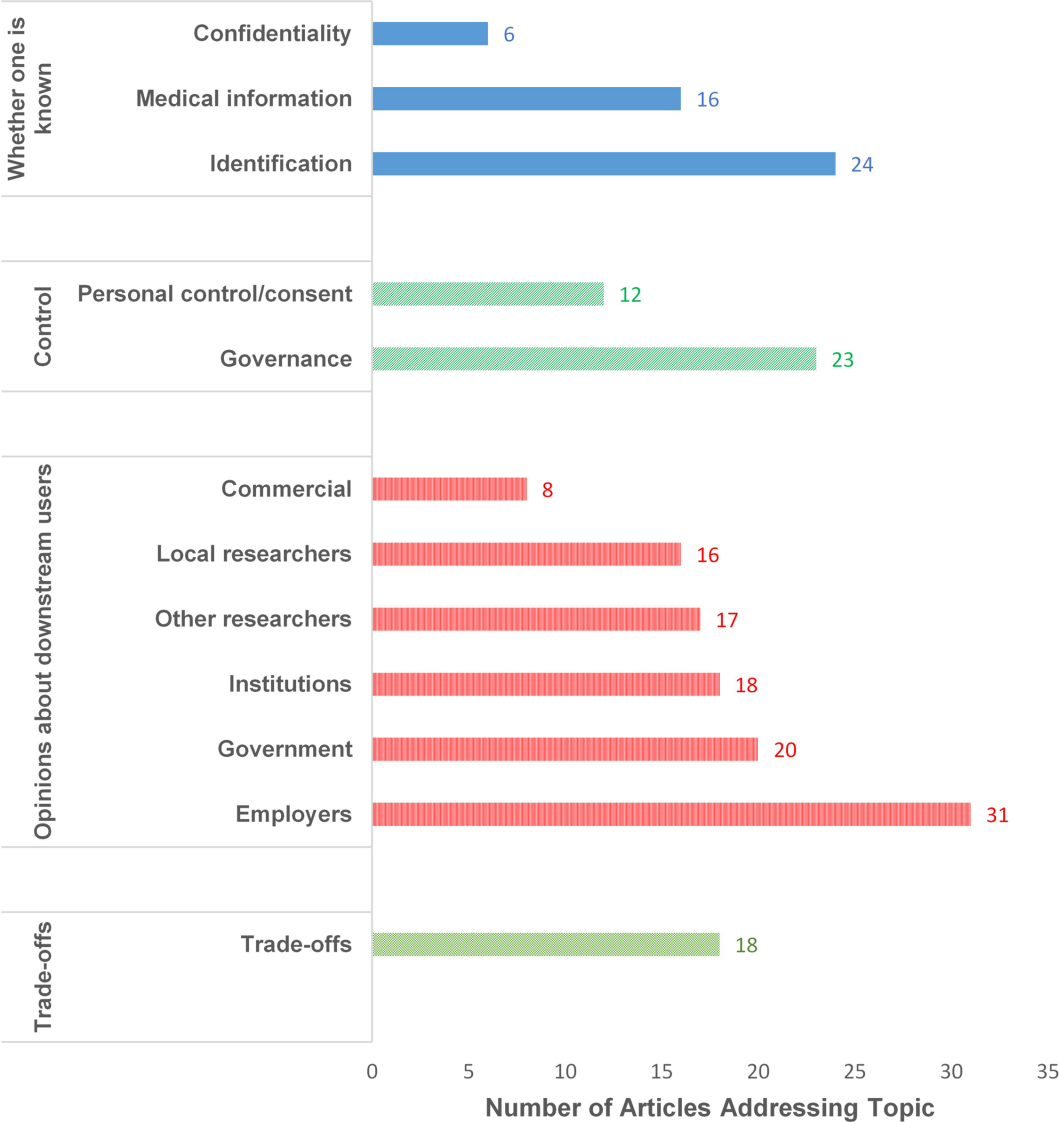
A summary of the number of articles that addressed each of the topics discovered in the systematic literature review.

As noted earlier, the investigators of this review developed a set of categories to apply to these studies, including whether one’s data or identity is known, control, downstream use and users, and tradeoffs between privacy and services or goods. Researchers varied widely in the specific issues they explored in their studies as described in Fig 3.

#### Whether one is known

**General concerns about privacy**. Investigators frequently asked about the general level of concern about privacy without eliciting further details about the respondents’ reasons for concern. [12, 19, 27, 29, 31, 35, 37–39, 44, 45, 49, 51, 59, 66, 70, 78] Some investigators focused solely on concerns about genomic information, [43, 54, 66, 79] while other researchers studying questions of privacy and genetics asked about the privacy of medical information generally either in lieu of, or in addition to, genetic data. ^,^[16, 28, 29, 42, 45, 52, 53, 56, 63, 67] For example, a survey of 13,000 patients at medical centers involved in the NIH-sponsored Electronic Medical Records and Genomics (eMERGE) consortium, which focused on consent and data sharing for genomics research, indicated that 11,397 (90%) agreed that “health information” privacy was important and that 8,135 (64%) were worried about this type of privacy. [12] In a mixed methods study conducted at Mt. Sinai Medical Center in New York City, 20 (57%) of the 35 healthy respondents who had undergone whole genome sequencing endorsed concerns about unspecified potential privacy issues in closed-ended questions. [34] A survey including 1,253 patients with bipolar disorder asked about “loss of privacy” among other issues. [49] A survey of 304 African-Americans in the Washington, DC, area revealed that 259 respondents (85%) were willing to undergo genetic testing for susceptibility to alcohol dependency, but 91 (30%) of participants had unspecified privacy concerns. Notably, these individuals indicated that, in general, they were more concerned about being labeled by their physicians than by family members. [19] Thirteen of 100 breast cancer patients (13%) at M.D. Anderson Cancer Center in Houston, Texas expressed significant concerns about a composite measure of “genomic data privacy.” [42] These studies, which all relied, at least in part, on surveys, may have prompted the respondents’ concern by their specific questions.

In some qualitative studies, few respondents volunteered worries about privacy when not specifically prompted. [36] In a study interviewing 65 parents of children being seen at primary care or multispecialty clinics at Vanderbilt University Medical Center, 21 (32%) endorsed privacy concerns when prompted, but none raised the issue on their own. [68] Similarly, a focus group study of women with breast cancer who had previously donated tissue at the Dana-Farber Cancer Institute similarly reported few privacy concerns—even when prompted—with two participants commenting that privacy protections might even hamper research. [71]

Concern that individuals might be identified was a commonly explored topic. [16, 27–29, 31, 35, 38, 52–54, 62–64, 72, 79–81] Almost half of the surveyed breast cancer patients at M.D. Anderson were concerned about having their name associated with their genomics results. [42] Additionally, some participants in the open access Personal Genome Project expressed mixed feelings about being identified even though they were told that their privacy could not be guaranteed. [62] By contrast, a number of studies reported that respondents were not particularly worried about the identifiability of their genetic information. [27, 30, 47, 50] Cancer patients in the Participant Issues and Expectations Project survey in Washington state were more worried about having their financial information stolen than their genetic data (69% vs. 3%). [30] Interestingly, only six studies explicitly explored whether people were worried about being re-identified from their DNA. [30, 31, 46, 47, 62, 81] In one study, parents were not worried about being re-identified from DNA because their affected children had a particular genetic syndrome with distinctive physical manifestations, such that they were under the impression that they were so readily identifiable anyway. [69] Far from desiring anonymity, some respondents wanted to ensure that they could be identified so they could receive research results. [29, 30, 34, 41, 79]

Studies typically revealed the fewest insights about reasons other than discrimination why people were worried that information about them would be revealed. In one study some respondents cited philosophical or religious reasons for their privacy concerns. [16, 56] A few studies examined both family and personal privacy. [32, 36, 37] Sometimes, concepts were conflated in ways that limited insight. In particular, privacy and confidentiality were often used interchangeably by both investigators and respondents. [32, 52, 73] As noted above, these refer to distinct and non-commensurate concepts. In other studies, these distinctions were made clear to the respondents. [33]

#### Control

**The roles of consent for and personal control of data**. Questions about various aspects of privacy were often explored in the studies that addressed acceptable approaches to informed consent and control of genomic data. [12, 18, 27, 28, 31, 33, 39, 42–48, 50, 52–54, 62, 63, 66–68, 71, 73, 74, 78, 82] For example, in one study in which IRB professionals, researchers, and participants were asked to identify which statements were most important to include in consent forms, opinions varied widely. The importance of getting consent for the release of identifying information was the only one endorsed in the top ten statements by all the respondents. [50]

Concerns about privacy were correlated with desires for more focused consent. In a study addressing consent for biobanking, those who believed that it is not possible to maintain their privacy and those who did not limit their Internet use because of privacy concerns were less likely to agree to broad consent for use of data about them with oversight for unspecified future research.[12] In one survey of a mixed group of stakeholders including participants in a cancer registry, their family members, and self-referred individuals with and without cancer, participants who perceived greater risks associated with genetic data were more likely to agree that re-consent to the use of data about them should be mandatory (p = 0.003). [27]

One particular question often raised in the context of consent was how much control participants should have over the downstream use of data and samples obtained from them. In a study interviewing 18 self-designated early adopters of genome sequencing and health devices (participants in the Personal Genome Project; attendees of the Genomes, Environments, and Traits conference; or participants in the Health Data Exploration project), all (100%) wanted control over their own data, including control over whether or not to authorize dissemination. [62] In a study interviewing parents of pediatric patients, family members and adult participants from cancer genomics studies (total n = 336), participants’ concerns included a lack of control over (mis)use and who could access their information in the public domain. [47] In a focus group study including 15 participants in a Baylor College of Medicine study of epilepsy gene resequencing, participants had mixed views regarding ownership and sharing of data. [81] Thirteen out of 15 (87%) participants wanted to maintain control over sharing, and most were concerned about privacy risks associated with public data release. Many opposed full public release of their DNA, and all (100%) opposed sharing with employers or insurance companies.

Notably, desire for ongoing control was not universal. Respondents in several studies were willing to cede decision making to IRBs and other oversight bodies. [28] This willingness to give up control was particularly evident in the studies demonstrating support for broad data sharing. [12, 13, 47, 52]

#### The roles of oversight, governance, and protection

Of the studies that explored views about the importance of governance, particularly regarding the use of data for research, some conflated oversight and security. [42, 52, 67, 74] In a focus group study (n = 49) including both participants in the NUgene biobank of Northwestern University Medical Center and the general public, roughly half of both groups (56% and 46%, respectively) were somewhat or very concerned about “[h]ow well … the privacy of individual genetic research data is protected.” Participants noted the importance of explicit privacy and security safeguards, oversight by a trustworthy institution, and penalties for misuse as important prerequisites for data sharing. [51]

Participants in several studies were skeptical that governance would be effective. [52, 62, 73] In a number of studies, some respondents simply felt that privacy could not be protected. In one nationwide online survey of English- and Spanish-speaking adults using Knowledge Network assessing views about genetic biobanks, of the 3,061 participants, roughly two-thirds, agreed that “maintain[ing] privacy is not possible” (1779 participants or 60%) or that privacy breaches are “inevitable” (1880 participants or 64%). Almost 1100 (37%) expressed concerns about the privacy of their medical information. [27] As we have demonstrated commonly occurs, some of these questions failed to distinguish between privacy and security.

While some studies revealed doubt about the efficacy of data protection by health care institutions, in a survey of 1,046 individuals who had purchased DTC genetic testing, most users had positive perceptions of privacy protections provided by the company. Specifically, 1004 individuals (96%) agreed that “In thinking about [the company’s] service, I am confident my privacy has been protected,” and 377 individuals (36%) felt that their test data was better protected than data in the medical record (398 individuals (38%) felt protections were similarly well protected; 41 individuals (4%) felt data were less protected: 241 individuals (23%) were unsure). [67]

Patients’ and the public’s views about the potential efficacy of legal protections were rarely probed. Of the 36 studies published since 2010—two years after the highly publicized enactment of the Genetic Information Nondiscrimination Act (GINA), which limited some uses of genetic data in health insurance and employment—only six reported individuals’ views about this law. [83] Individuals at risk for hereditary cancer participating in focus groups were skeptical of the protection provided by GINA, while researchers thought these patients were well informed, evidently thinking that patients should be reassured. [41] Some potential participants in the MedSeq project, which offered whole genome sequencing to healthy patients as well as those with cardiomyopathy as part of clinical care, were aware of the limitations of GINA, perhaps because these were addressed in the consent process. [63] In another study, 2,363 (79%) respondents from the general public agreed that this law made them “feel more comfortable that genetic information collected in the study could not be used against” them. [27] This finding led these investigators to recommend that biobanks clarify the limits of this law during the consent process. The potential protection provided by the Health Information Portability and Accountability Act (HIPAA), the Americans with Disabilities Act (ADA), bans on preexisting condition limits in health insurance, privacy torts, and state privacy laws were not reported at all.

#### Opinions about particular downstream users

A major concern expressed about identified genomic data was that some third party would misuse this information. Many studies, as noted below, examined respondents’ views—both positive and negative—about a host of downstream users. Often, these questions focused—at least in part—on whether downstream users might use these data in ways that the participants would not agree with or in a manner that would harm them. What opinions researchers are interested in has varied over time. For example, investigators have explored respondents’ concerns about use of genetic information by employers and insurers for the last thirty years, but they have probed views about government use of these data for only about the last decade.

#### Researchers, academic medical centers, and other nonprofit institutions in the United States

Investigators frequently asked specifically about how much people trusted researchers themselves as opposed to the entities in which they work. Respondents in 14 studies generally reported that they trusted researchers in the United States. [12, 30, 31, 33, 38, 42, 47, 48, 53, 56, 65, 69, 74, 79] Some participants expressed more trust in researchers in the institution where they seek medical care than they do in researchers at other academic institutions. [33, 42]

In eight studies, most participants expressed trust in their own health care institutions when asked, including patients who were cared for at eMERGE consortium academic medical centers (AMCs) as a group, [12] Group Health Cooperative of Puget Sound, [52, 67] M.D. Anderson, [42] Northwestern University Medical Center, [54] Dana-Farber Cancer Institute, [71] and Vanderbilt University Medical Center. [68] In a population-based survey of 639 individuals living in Nashville regarding BioVU, a biobank at Vanderbilt, 603 (88.8%) of respondents were somewhat or very confident that their “identity is protected when genetic information is used for research”, while 36 (11.2%) indicated that they were only a little or not at all confident. Additionally, 590 (93.9%) agreed that a DNA biobank is “fine as long as participants can choose whether to have their data included.” Similarly, 3,713 (92.2%) faculty and staff surveyed at Vanderbilt University were confident that the medical center adequately protected information. 3,766 (93.3%) endorsed the establishment and use of databanks when identifying information is removed; and 3,682 (91.6%) endorsed de-identified databanks, so long as research is approved by an ethics review board. [28]

In other studies, some participants expressed trust in AMCs and non-profits more generally. [41] In one study, only 20 of 100 (20%) cancer patients at M.D. Anderson were concerned about the privacy protection offered by research institutions. [42] Condit and colleagues [31] asked participants in the Northwest Cancer Genetics Registry how important trust in the researcher and the institution was to them, combining those ratings with answers about benefits and risks to create a composite trust score. Respondents in other studies, however, stated that they did not trust these institutions to protect their data. [43, 56] More than 11,000 (86%) respondents in a study of patients at the eMERGE institutions wanted to know if data were misused by researchers. [12] In another study with 931 U. S. veterans, almost all (97%) said that there should be a research agreement with the investigators with serious consequences in the event of breach. [29] Indeed, one study of users of DTC genetic tests found that this group trusted the companies more than they trusted their health providers and institutions to protect their information. [67]

#### Use by employers and insurers

The issue most frequently explored by investigators was participants’ worries about use of genetic and health information by insurers and employers. Of these, 31 reported that concerns about employers or insurers often were a significant concern. [12, 16, 18, 19, 28, 32, 35, 36, 38, 41, 43, 45, 47, 49, 50, 52, 54–56, 58, 59, 62–64, 66, 67, 70, 71, 74, 76, 79] For example, in a study of patients at a Northwestern University Medical Center-affiliated site who had contributed data to the NUgene biobank, roughly 30% to 60% of respondents identified potential risks of employer and insurance discrimination. [54] In a study interviewing 18 early adopters of genome sequencing and health devices, many were concerned about discrimination in insurance and employment. [62] Of 413 patients seen at Yale University outpatient cancer clinics who were asked about their interest in genomic tumor profiling, 198, 169 individuals (48,41%) were concerned about negative health or life insurance effects. [36] In a mixed methods study conducted at Mt. Sinai Medical Center, 10 of the 35 (29%) healthy respondents who had undergone whole genome sequencing worried about insurance discrimination. [34]

Evincing an even greater level of disapproval, participants in two studies reported that they thought that employers or insurers should not have access to genetic information for any purpose. In a survey of 1,046 individuals who had purchased DTC genetic testing, 1004 individuals (96%) of respondents felt that employers or insurers should not have access to the data. [67] Of the 931 U.S. veterans surveyed, 810 individuals (87%) felt it should be illegal for insurers or law enforcement to have access to the database. [29]

Other studies relayed that at least some of their respondents were not particularly concerned about whether their employers or insurers had access to their genetic information. [42, 68, 72] Some reported that their level of concern was mitigated to some extent either because 1) they were already sick or 2) they believed the nature of their job or social situation provided some protection.[63] Parents of pediatric patients, family members, and adult participants from cancer genomics studies (total n = 336) who had viewed an experimental consent form regarding public data sharing worried about identity theft and were more concerned about having their identity revealed than they were about health insurance discrimination. [38, 47, 48]

#### Commercial entities

Eight studies reported respondents’ thoughts about sharing their information with commercial entities, such as pharmaceutical companies. [30, 38, 43, 45, 47, 52, 58, 59, 72] For example, 44 of 100 (44%) patients at M.D. Anderson worried more about protection of privacy by drug companies than by research institutions or the government. [42] Respondents in another study, by contrast, felt that this was not a major reason for their decision not to take part in a biobank, [56] while participants in two other studies were confident that companies protected their privacy. [35, 67]

#### Government

Participants in 20 studies reported worries about providing access to genetic information to the government. [29–31, 35, 38, 40, 43, 46, 47, 52, 54–56, 58, 62, 67, 72, 73, 79] Use by law enforcement was specifically explored in two studies. One Michigan focus group study reported that some participants noted concerns about potential stigmatization by law enforcement. [45] In a particularly nuanced survey of 1,046 individuals who had purchased DTC genetic testing, 931 individuals (89%) felt it was very or somewhat “important that it be illegal for law enforcement to get their information.” [67]^(at 426)^

#### A gradient of trust in different users

Although one can infer from these studies that some genetic data recipients are seen to be more protective of their privacy than others, some investigators asked respondents to address this issue directly. [29, 52] One nationwide survey study, which defined privacy as “a condition where others have limited access to information about you” and security as “the protections that are in place to keep your information from being seen by people who do not have permission,” reported that of of the 1,319 respondents, 646 individuals (49%) did not trust researchers outside of the U.S. to keep their health information private or secure, while roughly 330 individuals (25%) did not trust U.S. researchers to do the same. [33] Rogith and colleagues assessed M.D. Anderson breast cancer patients’ willingness to share identified or de-identified genomic data under different conditions. [42] They reported the following gradient in respondents’ willingness to share that applied no matter whether or not the research offered direct benefit or used identified data: (physician involved in their care) > (physician at M.D. Anderson) > (any cancer researcher) > (any researcher) > (anyone).

#### The role of trade-offs–to what extent are people willing to forgo some privacy in order to receive other goods

Despite potential privacy concerns, many people were still willing to proceed with genetic testing in research or the clinic. [19, 29] A particularly detailed study interviewed parents of pediatric patients, family members, and adult participants from cancer genomics studies (n = 229) who had viewed an experimental consent form regarding public data sharing. Respondents stated concerns about having their identity revealed, “the lack of control over who could access their information in the public domain, fear of identity theft, anxiety about government access, apprehension over the potential commercialization of their DNA, and fear that their data would be used in morally objectionable research.” [47]^(at 109)^ The majority nonetheless endorsed both protecting privacy (84%) and advancing research (74%), and two-thirds indicated the advancement of research was the more important when forced to choose between the two options. Individuals enrolling in the MedSeq study expressed concerns about privacy, but were more confident in the study’s security and persuaded about the benefits of taking part. [63] In an earlier study interviewing African-American oncology patients, 34% of the 196 leaned toward enrollment in a genetic research study, even though they felt that their information would not be kept private. [65]

Some respondents were quite enthusiastic about participating and even impatient with slowing research. A focus group study of 26 women with breast cancer reported few privacy concerns even when prompted, with 2 (8%) participants commenting that privacy protections might hamper research. [71] One group of parents whose children had phenylketonuria (PKU) or leukemia felt that getting information was more important than protecting privacy. [39] Some participants offered strategies to reassure those considering genetic testing. In one focus group study including 35 North Carolina residents, respondents felt that privacy concerns about research participation could be mitigated by transparent communication about who would have access to their data and explicit security measures, particularly for how their blood would be handled. [64]

In other studies, however, worries about privacy led people to decide not to participate in research. [32] The main reason why 34 of 310 (11%) respondents invited to participate declined to enroll in the Kaiser Permanente Northwest biobank was concern about privacy and confidentiality. [56] Of the 173 (46%) of invitees who actively declined enrollment in MedSeq, 14 (8%) cited privacy concerns as a reason not to participate, including worries about hacking, security, and discomfort with having results in their medical record, while 48 (28%) cited fear of insurance discrimination and 22 (13%) worried about the psychological impact of the results. [63] Similarly, in one nationwide survey, most parents were interested in WGS of newborns, but the majority stated that the child’s privacy and the possibility of discrimination were very important. [66]

#### Drivers of privacy concerns

As we review below, most studies that reported information about potential drivers of concern assessed demographic factors such as race and age, attitudes toward risk or research, and trust orientation. Among studies evaluating demographic factors, non-White race and younger age were typically associated with greater privacy concerns or perceived risks. Associations of responses with other factors generally were not consistent.

#### Race/ethnicity

In almost all studies reporting differences in perspectives by race or ethnicity, non-White individuals had greater concerns about privacy, including more desire for control over use of their data and less willingness to share data than their White counterparts. In a survey including 1,253 patients with bipolar disorder that asked about “loss of privacy,” Blacks were more likely to be very concerned about loss of privacy as compared with Whites (41.9% vs. 24.6%, p < 0.0001). [49] In one study assessing perspectives about the use of residual bloodspots and including viewing of an educational movie about genetics research, African-American race, residence in Mountain states, and viewing an educational movie about retention and use of specimens were associated with a desire for notification about future use of the samples in research (p ≤ 0.05). [44] Among respondents willing to donate samples for genetic research (n = 1113) in another study, more African-Americans than Whites noted potential discrimination as a significant factor in reduced willingness to donate (64% vs. 36% White, p < 0.0001). [16, 53, 61] African-Americans were also less likely to accept broad consent vs. Whites (69% vs. 81%, p-value was not significant in adjusted analyses) in a study evaluating biobanking and consent preferences. [27] African-Americans were more likely to be unreceptive to genetic research (35.8% vs. 14.6%, p-value not reported), more likely to consider loss of confidentiality, genetic information being given to insurance companies, and genetic information-related discrimination to be major worries. [32] These reservations are not new, as African-Americans at the turn of the millennium were in one study more likely than Whites to have an initial negative reaction to news about the near-completion of the Human Genome Project (66.7% vs. 34.6% among Whites, p < 0.001) but not more likely to note privacy concerns. [74]

Non-White race was not a significant factor influencing privacy-related concerns in three studies. In one survey of 13,000 people, race and ethnicity were no longer statistically significant in determining views of data sharing once attitudes about research and biobanking were taken into account. [12] Asian and other ethnicities were associated with allowing use of residual newborn bloodspots in research. [44] In another, race and education were not statistically significantly associated with preferences for de-identification of samples stored in a cancer tissue biobank. [70]

Studies assessing perceptions related to clinical genetics similarly reported more privacy-related concern among Non-White participants. Latinas in one study were most likely to agree that genetic testing results would not stay confidential. [73] African-American and Latina participants were also more likely to worry that genetic testing results would be used to show that their ethnic group is not as “good” as others (p < 0.03). African-Americans had the highest level of medical mistrust on a 60-point scale (mean of 29.2 vs. 27.3 for Latinas and 19.4 for Caucasians, significantly higher in African-Americans and Latinas vs. Whites, p ≤ 0.0001). In one study, African-Americans and Hispanics were less likely to say they would participate in pharmacogenomic testing than Whites or Asians (p < 0.05). [72] Conversely, if disclosure of pharmacogenomic testing results to employers were to occur, African-Americans (Odds Ratio (OR) = 1.88, p ≤ 0.01), Asians (OR = 2.6, p ≤ 0.001), and Hispanics (OR = 3.1, p ≤ 0.001) were more likely than Whites to participate in genetic testing. [72]

#### Age

Several studies also addressed the effects of age on differences in perceptions, with older people typically endorsing fewer privacy-related concerns. In one study, younger veterans and those serving in or since the Vietnam War considered privacy important in the design of a biobank. [29] In another study, older participants were not as likely as younger ones to feel that personal identification from shared data was an imminent risk. [38, 47, 48] In a third study, older individuals participating in a study of dementia risk had less concern about sharing genetic their genetic data in publicly accessible databases, although most participants, regardless of age, felt that data sharing benefits outweighed the risks. [52] In a study of pharmacogenomics testing, individuals over age 50 expressed less concern about anonymity than younger individuals. [72] Another study noted that younger people were more likely to want notification about the use of newborn blood spots for research. [44] Older people were not, however, completely sanguine about this type of research. In a final study, older respondents were more likely to endorse the removal of identifiers if samples were to be used in future research (p ≤ 0.009). [70]

#### Sex

The influence of a respondent’s sex on privacy opinions was not demonstrated consistently. In one study, female sex was associated with allowing use of residual newborn bloodspots in research, [44] while in another, women were more likely than men to find narrow consent for biobank research acceptable (p ≤ 0.01). [27] A third study reported few differences between male and female willingness to participate in pharmacogenomics research, [72] and while another reported little difference in the level of privacy concern among male and female respondents to questions about participating in psychiatry-related genetic research. [49]

#### Status as a parent

The demonstration of parental views on genetic privacy has similarly been mixed. In one study, the parents of pediatric patients selected more restrictive data sharing options than older participants (OR for selecting no release: 6.88, 95% Confidence Interval (CI): 2.19 to 21.61). [38, 47, 48] More parents than other adults felt it was extremely important to be involved with data sharing decisions (OR = 4.11; 95% CI: 1.67–10.12, p ≤ 0.006). By contrast, in a different study, parents with a higher education level, youngest child with multiple health conditions, or those planning to have a child in 5 years were more interested in newborn WGS, even when future use of the data was a possibility (OR = 1.81–1.95, p ≤ 0.05). [66] In another study, significantly more individuals without living childen noted that they were very concerned about privacy than those with children (29% vs. 25%, p ≤ 0.04). [49]

#### Education and socioeconomic factors

Privacy concerns were not consistently associated with either higher or lower levels of education or income. In a large survey (n = 13,000), individuals with higher education were more likely to express willingness to provide broad consent for data sharing. [12] In one biobanking study, respondents with a higher education level and those with a higher household income were more likely to consider privacy important. [29] Similarly, individuals with higher education and income levels were more willing to participate in pharmacogenomic testing if data collected were “anonymous” and not disclosed to employers or insurers. [72] In another study of African-Americans’ willingness to enroll in a cancer genetics registry, higher education was associated with enrollment, while lower education was associated with lower trust. [65] In one survey of 1,243 participants in the US Bipolar Genome Study assessing psychiatric genetic research, 410 individuals (33%) of people with a disability, 336 individuals (27%) of those who were unemployed, and 224 individuals (18%) of respondents in managerial positions were very concerned about privacy (p ≤ 0.02). [49]

#### Other modifiers of perceptions

The studies addressed a number of other factors that modified attitudes toward privacy. In one large survey, people who were more religious were less likely to support broad consent for data sharing. [12] Another study reported that participants with “conservative political views” were less likely to endorse WGS than those identifying as politically moderate (OR = 0.64, p ≤ 0.05). [66] In a study of African-Americans’ opinions about a cancer genetics registry, less satisfaction with one’s cancer care and an individualistic versus collectivist (family) orientation were associated with lower trust (p ≤ 0.02). [65] Cancer patients in a different study categorized as having a high conversation orientation, in which the family climate encourages free interaction (as opposed to a conformity orientation, in which family climate stresses homogeneity of attitudes and beliefs) had more permeable family and privacy boundaries and were more likely to communicate about genetic cancer risk with family members. [37]

### Views of professionals

Fewer studies explored the views of professionals. Some studies included mixed stakeholder groups or non-patient/public stakeholders. In one study in which IRB professionals, researchers, and participants were asked to identify which statements were most important to include in consent forms, the opinions varied widely. The statement that “there is a risk that someone could get access to data we have stored about you,” as well as three statements about privacy protections were deemed important more often by researchers and IRBs than by research participants. [50]

IRB professionals generally were more likely than investigators to worry that research participants would be identified and come to harm. In a study comparing views of genetics researchers and IRB personnel, more researchers than IRB personnel felt that personal identification was unlikely to result from a study involving coded genetic data (70% vs. 52%). [46, 60, 77] Similarly, few researchers felt harm was likely to result from identification (11%) compared with 37% of IRB professionals.

In a twenty-year-old study assessing the views of journal editors and genetic researchers, of the 177 researchers surveyed, 90 individuals (51%) had confidentialityconcerns about publishing genetic pedigrees. [75] Four of the 14 (29%) editors surveyed noted that they had been contacted by a research participant about perceived breaches of privacy or confidentiality, while eight had confidentiality concerns about publishing pedigrees (while two had no concerns and four were not certain).

## Discussion

Making genomic and affiliated data available for research is a critical goal for various policies in the United States, one that many people endorse. [13] The NIH, under its Genome Data Sharing Policy, requires its investigators to get broad consent for data sharing as a condition of funding for genome-wide research and mandates that identifiers be removed from data prior to being shared or deposited into databases for use by other investigators. [9] Current laws, such as the Health Insurance Portability and Accountability Act (HIPAA), [84] permit the sharing of patient information, at times in identifiable form, without permission of the individuals to whom the data corresponds, in a variety of circumstances including for some forms of research. [85] The U.S. Common Rule also permits the use of de-identified data without consent and with limited to no IRB oversight and endorses an expansive role for broad consent of identified data. [86] Yet policies and laws must ensure that adequate attention be paid to the concerns, many of which fall under the broad term “genetic privacy,” of those from whom data are obtained to sustain public support for genomic research. A critical step, then, is to ascertain what apprehensions people have about genetic privacy and the use of data about them.

The picture of genetic privacy that emerges from this systematic literature review is complex and riddled with gaps. When asked specifically “are you worried about genetic privacy,” the general public, patients, and professionals frequently agreed. In many cases, however, that question was posed poorly or only in the most general terms, leaving the reader and policy makers with little idea of what specific issue or issues actually concerned respondents. In the future, investigators need to specify which particular aspects of genetic privacy they are interested in. Since decisions about genetic testing and data are not made in a vacuum, it is critical to ask respondents about what trade-offs they are willing to make and risks they are willing to accept for their own health, for advancing science, and for their interest and convenience.

A number of the studies we examined did provide some insight into what worries people have. Many participants expressed concern that genomic and medical information would be revealed to others. Yet rather than parsing out how participants thought these revelations might occur, investigators and respondents frequently seemed to conflate privacy, confidentiality, control, and security. [78] Although the term *confidentiality* was used quite frequently, none of the studies explored participants’ beliefs about whether clinicians would breach such an obligation. This was true even for the few studies that focused on patients’ expectations and concerns about clinical genetic testing.[36, 42, 63, 69] Understanding what patients think clinicians will do with genomic data warrants more attention for a number of reasons. The first is the ongoing debate about whether physicians should warn at-risk relatives–even over the initial patient’s objections– of genetic findings that have implications also for them. [87, 88] More generally, worries that identified genomic information may be revealed in the setting of clinical care may have some merit since genomic information—some of which comes from return of research results—is increasingly incorporated into electronic medical records, where many security breaches have transpired. [89] Little research has been done, moreover, to understand what people think about the revelations inherent in the personal health information that they are often compelled to provide [90] or the impact of their participation or that of their relatives in direct-to-consumer testing on the availability of data that also have implications about their health. [91, 92] Interestingly, at least some who place their personally identified information in open access databases nonetheless expect their privacy to be protected. [18]

By contrast, despite references by the NIH [93] and in the proposed changes to the Common Rule [94, 95] to the case of Henrietta Lacks, whose cervical cancer cells were used to create a widely used cell line, [96] to the best of our knowledge, there have been almost no reports of breaches of research data and few reports of deliberate efforts to identify research participants except those undertaken for demonstration. [97–99] Still, it is important to understand the types of data-driven attacks that individuals could perpetrate against genomic data. First, it has been demonstrated that less than one hundred genomic variants, in the form of single nucleotide polymorphisms, are sufficient to represent an individual uniquely. [100] Thus, when someone has an identified genomic record, then matching it to a de-identified record with the same variants would be relatively trivial. It has further been shown that a de-identified genomic record can be linked back to an individual’s identity through inference strategies. For instance, Gymrek and colleagues showed that patterns of short tandem repeats (STRs) on the Y-chromosome could be used to infer the surname for the corresponding individual through online websites where <surname, STR> information are reported. [98] And in the event that additional demographics are available on the corresponding person (e.g., geographic information in the form of U.S. State of residence and age), the record could be uniquely linked to data on named individuals gathered from information brokers.

While these identifications were committed against individual-level genomic records, it has also been shown that an individual’s presence can be detected in summary genomic data from genome-phenome association studies. This was initially demonstrated by Homer and colleagues, where they showed that, if a named genomic record was in hand, then the similarity of that record could be compared to the aggregate statistics from a study and some reference population, such as 1000 Genomes. [97] This attack required on the order of 10,000 genomic variants, but it this type of “presence” attack has been subsequently been refined over the years to require less genomic information [101] and adapted to penetrate the Beacon system of the Global Alliance for Genomics and Health. [102] Yet it has also been shown that such attacks may make overly strong assumptions about the prior probability that a targeted individual could be in the study to begin with, which suggests that the chance of a privacy violation is much lower than was previously thought. [103] This concept was formalized in a game theoretic framing of the problem, where it was shown that the amount of effort and expense necessary to perpetrate an attack may be greater than the actual value one reaps from doing so, [104] suggesting that while attacks on genomic data are possible, individuals and entities in the private sector may not be likely to attempt them.

Nonetheless, there has been much debate about the risk that a person could be re-identified from their de-identified DNA or genetic data and many calls for more robust protection. [105–108] The identifiability issue, for example, animated recent proposals to designate biospecimens and data derived from them as identifiable *per se* for purposes of human research regulation. [94, 95] In 2015 the Working Group of the Precision Medicine Initiative went further and recommended the creation of additional safeguards for data and legal penalties for inappropriate re-identification. [109] Under the authority of the 21^st^ Century Cures Act, the NIH now automatically issues Certificates of Confidentiality to all federally-funded research involving biospecimens and individual genomic data. [110] Yet one of the main reasons the drafters of the proposed changes to the Common Rule cited for not definining biospecimens as identifiable, which would have required almost universal consent, was that researchers and particularly the public worried that such a provision “could significantly harm the ability to do important research without producing any substantial off-setting benefits.” [111] Even more telling, when explicitly asked, people frequently opined that they were willing to pursue genetic testing in the clinic or in research even with some risk to their privacy.

Some investigators, as part of their exploration of privacy, explicitly asked how much control people wanted to have over the use of the genomic data they had provided. The responses varied widely from participants' being happy to cede control to others to their wanting the ability to decide about all future uses. This range was similar to those seen in examinations of individuals’ opinions about data sharing more broadly, which often varied depending on context, such that parents were often more willing to share data about themselves than that of their children. [12, 13]

Many of the studies we examined focused largely on assessing individuals’ worries that they could be harmed if genetic information were divulged. In particular, concern about adverse use by employers and insurers loomed large, both for investigators and the public, especially taking into account the 149 papers that were excluded from this analysis because they focused solely on discrimination. Given the amount of attention devoted to eliciting respondents’ worries about being harmed by how others use genetic data, it is noteworthy that of the studies we examined, few studies explored patients’ and the public’s opinions about the efficacy of legal protections against discrimination in employment and access to health insurance and of privacy more generally. The few that did focused on GINA, which limits how genetic information can be accessed and used. Some respondents found this law reassuring, although others noted the strong criticisms that have been leveled against it for its lack of efficacy. [112, 113] The potential value of other pertinent laws, such as HIPAA, the Americans with Disabilities Act, and those that eliminate the exclusion of pre-existing conditions from coverage in health insurance, was not explored at all—even though all of them offer some degree of protection against discrimination on the basis of genetic information. [114] Ultimately, moreover, there is little evidence that employers and insurers actually engage in discrimination on the basis of genetic information. [22] More generally, none of the studies we examined explored whether participants felt that they were protected by common law privacy claims or state privacy laws. While we recognize that investigators are limited in the number of topics they explore, it is nonetheless striking how little is known about people’s opinions about how well the law protects them and information derived from them. Although legal scholars and advocates have devoted much attention to these strengths and weaknesses of these legal protections,[115, 116] it would be helpful to know more about how much these laws matter to patients and research participants, particularly as they make decisions about clinical testing and research participation.

Participants frequently expressed concerns about how the government might use data about them. These individuals tended to fall into two main groups. Many opined that government databanks simply would not provide adequate security for these data so that information about the participants would end up in the wrong hands. Others expressed concern that law enforcement would access these data, often for criminal investigations, particularly if the data were already held by the government. These studies, of course, were conducted prior to the use of information contained in GEDMatch, a genealogy database, to identify the Golden State Killer,[117] which has spurred a new debate about whether it is appropriate for the government to use such data to identify potential perpetrators.[118] Interestingly, genomic data collected in federally funded research is particularly protected from such uses since “identifiable, sensitive information,” which was defined by the NIH in its recent guidance on Certificates of Confidentiality as including “[r]esearch that involves the generation of individual level, human genomic data from biospecimens, or the use of such data.,”[110] is “immune from the legal process, and shall not, without the consent of the individual to whom the information pertains, be admissible as evidence or used for any purpose in any action, suit, or other judicial, legislative, or administrative proceeding.”[119]

The research conducted to date indeed sheds some light on which people are more likely than others to have concerns about privacy. This is important because there is much reason to pursue the involvement of all people in genomics research in order to discover the impact of genomic variation on disease and health and to be concerned about the availability of genomic medicine for underrepresented populations due to inadequate inclusion in research. [120] Thus, the fact that more racial and ethnic minorities in many studies express concerns about the privacy of their genetic information and reluctance to take part in research than do Whites makes the challenges of addressing the trepidations of minority populations clear. At the same time, it is also important that some people have reservations about genomic information among all demographics studied.

Much remains to be learned about the factors that form people’s beliefs about genetic privacy, including their family and community networks, in person and virtual, as well as media, TV, film, and popular culture. [121, 122] We believe it is important for future studies to conduct investigations at greater depth into which concerns about genetic privacy are most salient to people, the social forces that influence those perceptions, and the contexts that affect their decision making. It is further critical to identify the social practices that will make the collection and use of these data more trustworthy for participants and patients, as well as to identify the circumstances that lead people to set aside worries and decide to participate in research. Even though not all people will seek or endorse the collection and use of genomic data, a more holistic understanding of the social practices and structures in which these data are obtained can enable individuals, groups, and policy makers to make more informed choices in the future.

It is nonetheless possible, based on our analysis of existing data, to make some observations and recommendations about current consent practices. The new provisions for broad consent for use of identifiable private information or identifiable biospecimens do require the disclosure of information about who may have access to data about research participants. [123] ^(at §__.116(d))^ More, however, needs to be disclosed about the likelihood that an individual could be harmed by third parties depending on who is using the data. In particular, access by non-researchers to de-identified data used in federally-funded research is actually quite limited legally and practically. By contrast, returning personal research results to participants, especially if those data are placed in their medical records or posted to open access webistes, opens these individuals up to greater risk, both because such results are more accessible to others and because the law’s protection against adverse use once data are available is incomplete.

## Materials and methods

### Information sources and eligibility criteria

To perform this review, we searched multiple databases across various disciplines, including the MEDLINE database via PubMed, Web of Knowledge (Science Citation Index and Social Sciences Index), Applied Social Sciences Index, PsycInfo, ACM Digital Library, IEEE Explore, and Sociological Abstracts from January 1990 to November 2016. We applied a combination of controlled vocabulary and key terms related to genetics, privacy, genetic research, DNA databases, and perception (e.g., “privacy”, “confidentiality”, “genomics”, and “opinion”). In addition, we hand-searched the reference lists of included articles and recent reviews [13, 124–129] addressing privacy and genetic information to identify additional potentially relevant articles. This hand-searching process continued until July 2017. The complete set of search strategies is available in S3 Table.

We developed inclusion criteria collaboratively, including empirical studies of any design that contained primary data about individuals’ opinions or perceptions related to real or hypothetical privacy issues associated with human genetic information in clinical care and research. We limited inclusion to English language studies conducted solely with U.S. populations. We used a semi-automated screening process to conduct an initial screen of the titles of abstracts of studies to eliminate non-empirical research. The screening algorithm was architected to search for investigations where 1) the country in which a study was run (to triage studies outside of the United States), 2) the focus was solely on non-human genetics (e.g., genetically modified crops and animals), and 3) no empirical results were generated. We further searched the titles, abstracts, and indexing terms and controlled vocabulary of the group of “non-empirical” studies culled by the algorithm for the use of salient keywords, such as privacy or confidentiality. In this latter case, we retained these studies for manual screening as described below. Two investigators (EC, BM) independently screened 2,335 studies classified as an empirical investigation for inclusion, with disagreements resolved through discussion to reach consensus. We excluded 149 articles that addressed only views about genetic discrimination since that topic was not the primary focus of this review. Ultimately, 53 studies were included for further analysis.

### Assessment of study quality

Four investigators (EC, CH, BM, NS) independently evaluated the methodologic quality of studies using prespecified questions employed in a prior systematic review of informed consent for biobanking. [13] Two reviewers (EC, NS) resolved discrepancies in quality assessment.

### Data extraction and analysis

One team member (NS) initially extracted the study design, study population characteristics (e.g., age, sex, and stakeholder type), methodologic characteristics (study design), and baseline and outcome data on constructs of interest (privacy-related perceptions) from eligible studies. Characteristics of studies identified later were extracted by three other team members (EC, CH, SS). The findings were too heterogeneous to permit a meaningful quantitative analysis.

### Categorizing aspects of privacy explored in these studies

Three investigators (EC, CH, SS) analyzed each study in order to determine which aspects of privacy the investigators explored and, in the case of qualitative studies, which topics participants raised. These team members developed categories for these aspects of privacy through an iterative process informed by categories raised in earlier literature, illustrated in Fig 1. The first set of topics–whether data or identity are known, which often elicits dignitary concerns–was subdivided into concerns about 1) identifiability, 2) confidentiality, and 3) other issues, such as self-identity. We grouped articles addressing informed consent and the desire for personal control to protect privacy with the value of institutional practices and laws in providing protection into the domain of control. The next domain–data users–addressed the various entities who potentially could use data, which included researchers, academic institutions, commercial entities, government agencies, and foreign users. For each entity in this group, we examined responses regarding fears and risks, on the one hand, and trust, on the other. Finally, we ascertained whether investigators explored participants’ views about their willingness to trade their own privacy for some service or good, such as advances in science. Two team members (EC, CH) then labeled each study with these categories. Some of the individual studies addressed more than one aspect of privacy. Disagreements were resolved through debate to reach consensus.

## Supporting information

S1 Table List of studies included in the systematic literature review with first author, year of publication, population studies, research method(s), number of participants, and categories of concerns regarding genetic privacy assessed. (DOCX)

S2 Table Main data extraction files. (XLSX)

S3 Table GetPreCiSe search strategy. (PDF)
