# Supplementary material for: A systematic literature review of individuals’ perspectives on privacy and genetic information in the United States
Source: PLoS One. 2018 Oct 31;13(10):e0204417. doi: 10.1371/journal.pone.0204417 (PMC6209148; doi:10.1371/journal.pone.0204417)
Supplement: S1 Table — (DOCX) [file pone.0204417.s001.docx]

**S1 Table. List of studies included in the systematic literature review with first author, year of publication, population studies, research method(s), number of participants, and categories of concerns regarding genetic privacy assessed.**

| **First author of study** | **Year** | **Population** | **Methods** | **Number** | **Dignitary Interests** | **Consent/**  **Governance** | **Downstream Users** | **Trade-Offs** | **Unspecified Privacy** |
| --- | --- | --- | --- | --- | --- | --- | --- | --- | --- |
| Haeuser-mann | 2017 | DTC | Survey | 550 | X | X | X | X |  |
| Sanderson | 2017 | General public | Survey | 13,000 |  | X | X |  | X |
| Goodman | 2017 | Patients, relatives | Survey | 450 | X |  | X | X |  |
| Condit | 2016 | Cancer registry | Survey | 450 | X | X | X | X | X |
| Cheung | 2016 | General public | Interview | 18 | X | X | X | X |  |
| Majumder | 2016 | General public | Survey | 1319 |  | X | X |  |  |
| Weidman | 2016 | General public | Survey | 484 | X |  | X | X | X |
| Yushak | 2016 | Oncology patients | Survey | 415 | X |  | X |  |  |
| Freeman | 2016 | Parents/ caregivers | Survey | 439 +192 |  |  | X |  |  |
| Sanderson | 2016 | Patients | Interview | 35 |  |  | X |  |  |
| Robinson | 2016 | Patients/ consumers, primary care/ cardiology | Interview | 202+173 | X | X | X | X |  |
| Rauscher | 2015 | Cancer risk patients | Survey | 175 | X |  |  |  | X |
| Ewing | 2015 | General public | Survey | 3061 | X | X |  |  | X |
| Burstein | 2014 | Children with cancer, adults in a cancer study | Interview | 309 | X | X | X | X | X |
| Scott | 2014 | General public | Questionnaire | 304 |  |  | X |  | X |
| Nagaraj | 2014 | Parents of children with PKU/  Leukemia | Survey | 49 | X | X | X |  | X |
| Goldenberg | 2014 | Parents/ caregivers | Interview | 1539 | X | X | X |  | X |
| Rogith | 2014 | Patients | Survey | 100 | X | X | X |  |  |
| Quinn | 2014 | Patients in waiting, researchers | Focus group | 40 +32 | X | X | X |  |  |
| Bollinger | 2013 | DTC | Survey | 1046 | X | X | X |  | X |
| Platt | 2013 | General public | Survey | 1704 + 1643 | X | X | X |  | X |
| Thiel | 2013 | General public /NBS | Commun-ity meetings | 393 | X | X | X | X |  |
| Duquette | 2012 | General public | Focus group, survey | 87 | X | X | X | X |  |
| Botkin | 2012 | General public, NBS | Mixed methods | 3855 |  | X |  | X | X |
| Tabor | 2012 | Parents/ caregivers | Interview | 9 | X |  |  | X |  |
| Brothers | 2012 | Patient/ consumer | Interview | 65 | X | X | X |  |  |
| Lemke | 2012 | Patients/ caregivers | Focus group | 45 | X | X | X | X | X |
| Oliver | 2012 | Research participants | Mixed | 229 | X | X | X | X |  |
| Edwards | 2012 | Researchers and IRB | Survey | 559 | X | X | X |  |  |
| Brothers | 2011 | General public | Survey | 4050 | X | X | X |  | X |
| McGuire | 2011 | Patients/ guardians | Mixed | 336 |  | X | X |  |  |
| Nwulia | 2011 | Research participants | Survey | 1253 | X |  | X |  | X |
| Lemke | 2010 | IRB | Survey+ interviews | 208+31 | X |  |  |  | X |
| Trinidad | 2010 | Participants, surrogates | Focus group | 79 | X | X | X | X |  |
| Beskow | 2010 | Public/IRB/researchers | Interview | 40 | X |  |  |  |  |
| Beskow | 2010 | Researchers and research personnel, patients/ consumers | Interview | 84 | X | X | X |  |  |
| Goddard | 2009 | Patients | Survey | 500 | X | X | X |  | X |
| Goldenberg | 2009 | Patients at AHC | Interview | 1193 | X | X | X |  |  |
| Ormond | 2009 | Patients/consumers | Interview | 109 | X | X | X |  |  |
| Kaufman | 2009 | Veterans | Survey | 931 |  | X | X | X | X |
| Hull | 2008 | Patients | Survey, interview | 1193 | X | X | X |  | X |
| Skinner | 2008 | Patients/ consumers | Interview | 262 | X | X | X |  |  |
| McGuire | 2008 | Research participants | Focus group | 15 | X | X | X | X |  |
| Leiman | 2007 | HCPs | Survey | 41 | X | X | X |  |  |
| Helft | 2007 | Patients | Survey | 273 |  |  | X | X | X |
| Kaphingst | 2006 | Patients/ consumers | Interview | 26 | X | X | X | X |  |
| Freeman | 2006 | Surrogates | Interview | 146 | X | X | X |  |  |
| Bevan | 2003 | General public | Focus groups | 102 |  |  | X |  | X |
| Rothstein | 2003 | General public | Survey | 1796 | X |  | X |  |  |
| Thompson | 2003 | Parents/ caregivers | Interview | 273 |  | X | X |  |  |
| Tambor | 2002 | General public | Focus group, survey | 407 | X | X | X |  |  |
| Wertz | 1998 | General public | Survey | 476+ 988 |  |  | X |  |  |
| Botkin | 1998 | Researchers and journal editors | Survey | 191 | X |  |  |  |  |
