# Supplementary material for: A systematic literature review of individuals’ perspectives on privacy and genetic information in the United States
Source: PLoS One. 2018 Oct 31;13(10):e0204417. doi: 10.1371/journal.pone.0204417 (PMC6209148; doi:10.1371/journal.pone.0204417)
Supplement: S3 Table — (PDF) [file pone.0204417.s003.pdf]

S2Table. GetPreCiSe Search Strategy

Databases

- ACM Digital Library
- AnthroSource 2.0 (American Anthropological Association)
- IEEE
- PubMed
- Web of Science (Social Sciences Index, Science Citation Index)
- Applied Social Sciences Index and Abstracts (ASSIA)
- International Bibliography of the Social Sciences (IBSS)
- PRISMA Database with HAPI Index
- PsycARTICLES
- Psychology Database
- PsycINFO
- Public Health Database
- Research Library
- WorldCat

Hand searching

- Reference lists of recent, relevant systematic reviews and comprehensive narrative reviews
- Reference lists of publications included after the full-text review
- Table of Contents from relevant journal titles
- Publications from key authors

Journals

- BMC Medical Ethics
- Genetics in medicine: official journal of the American College of Medical Genetics
- Public Health Genomics
- Journal of Medical Ethics
- American Journal of Bioethics
- The Journal of law, medicine & ethics: a journal of the American Society of Law, Medicine & Ethics
- Critical Studies in Media Communication
- Communication and Critical/Cultural Studies
- Communication, Culture, & Critique
- Journal of Health Communication.

Other

- American College of Medical Genetics and Genomics Publications (https://www.acmg.net)
- G2P Research in Translational Genomics and Health Outcomes (http://www.genomes2people.org/home)
- EthicShare (http://ethicshare.org)
- HumGen International Database (http://www.humgen.org)
- PopGen Database (http://www.popgen.info/home)

Search Strategy: PubMed

**Date**	5/18/2016
**Platform**	PubMed
**Databases**	Medline
**Notes**	Preliminary, scoping

Search	Strategy	Records
1	Search perception[tiab] AND genetics AND ethics	70
2	Search ("Attitude"[Mesh]) OR "Public Opinion"[Mesh]	300,753
3	Search "Biometric Identification"[Majr]	4,858
4	Search ("Biometric Identification"[Mesh]) AND (("Attitude"[Mesh]) OR "Public Opinion"[Mesh])	20
5	Search ("Privacy"[Mesh]) OR "Confidentiality"[Mesh]	54,296
6	Search ((("Privacy"[Mesh]) OR "Confidentiality"[Mesh])) AND "Biometric Identification"[Majr]	130
7	Search ((("Privacy"[Mesh]) OR "Confidentiality"[Mesh])) AND ("databases, genetic"[mesh] OR "dna fingerprinting"[mesh]) Schema: syn	643
8	Search "Perception/ethics"[MeSH Terms]	31
9	Search ("Public Opinion"[MAJR]) AND "Genetic Research"[MAJR]	32
10	Search ((("Privacy"[Mesh]) OR "Confidentiality"[Mesh])) OR (("Attitude"[Mesh]) OR "Public Opinion"[Mesh])	349,196
11	Search (("databases, genetic"[mesh] OR "dna fingerprinting"[mesh])	48,693
12	Search "databases, genetic"[mesh] OR "dna fingerprinting"[mesh]	48,693
13	Search "Biometric Identification"[Mesh]	13,594
14	Search ("Biometric Identification"[Mesh]) OR ("databases, genetic"[mesh] OR "dna fingerprinting"[mesh])	49,106
15	Search Genetic Research"[Mesh]	252
16	Search (("Genetic Research"[Mesh]) OR (("Biometric Identification"[Mesh]) OR ("databases, genetic"[mesh] OR "dna fingerprinting"[mesh])))	56,129
17	Search ((("Surveys and Questionnaires"[Mesh]) OR "Attitude"[Mesh]) OR "Public Opinion"[Mesh]) OR "Informed Consent"[Mesh]	1,014,394
18	Search ((((("Surveys and Questionnaires"[Mesh]) OR "Attitude"[Mesh]) OR "Public Opinion"[Mesh]) OR "Informed Consent"[Mesh])) AND ((("Genetic Research"[Mesh]) OR (("Biometric Identification"[Mesh]) OR ("databases, genetic"[mesh] OR "dna fingerprinting"[mesh]))))	1,395
19	Search ((((("Surveys and Questionnaires"[Mesh]) OR "Attitude"[Mesh]) OR "Public Opinion"[Mesh]) OR "Informed Consent"[Mesh])) OR (("Privacy"[Mesh]) OR "Confidentiality"[Mesh])	1,052,129
20	Search (((((("Surveys and Questionnaires"[Mesh]) OR "Attitude"[Mesh]) OR "Public Opinion"[Mesh]) OR "Informed Consent"[Mesh])) OR (("Privacy"[Mesh]) OR "Confidentiality"[Mesh]))) AND ((("Genetic Research"[Mesh]) OR (("Biometric	2,277

	Identification"[Mesh]) OR ("databases, genetic"[mesh] OR "dna fingerprinting"[mesh]))))	
21	(((((((("Surveys and Questionnaires"[Mesh]) OR "Attitude"[Mesh]) OR "Public Opinion"[Mesh]) OR "Informed Consent"[Mesh])) OR (("Privacy"[Mesh]) OR "Confidentiality"[Mesh]))) AND ((("Genetic Research"[Mesh]) OR (("Biometric Identification"[Mesh]) OR ("databases, genetic"[mesh] OR "dna fingerprinting"[mesh])))))) OR (perception[tiab] AND genetics AND ethics)	2,335

Search Strategy: Web of Science

**Date**	5/18/2016
**Platform**	Web of Science
**Databases**	SCI-EXPANDED, all years
**Notes**	Preliminary, scoping

Search	Strategy	Records
1	TS=(perception OR perceive OR opinion OR attitude OR fear OR belief OR view) AND TS=(genetic OR biobank OR DNA OR genome) AND TS=(ethics OR sociology OR consent OR privacy OR confidentiality OR identity OR autonomy)	1,950
2	TS=public survey AND TS=genetic	739
3	TS="public perception" AND TS=genetic	53
4	TS=("perception" OR "public opinion") AND TS=(genetic OR genome OR DNA) AND TS=(ethic* OR privacy)	115
5	TS="human genome" AND TS="public perception"	3
6	TS=”belief AND TS=”participating” AND TS=”genetic”	81
7	#1 OR #2 OR #3 OR #4 OR #5 OR #6	2,725

Search Strategy: ProQuest

**Date**	6/16/2016
**Platform**	ProQuest
**Databases**	Applied Social Sciences Index and Abstracts (ASSIA), International Bibliography of the Social Sciences (IBSS), PRISMA Database with HAPI Index, PsycARTICLES, Psychology Database, PsycINFO, Public Health Database, Research Library
**Notes**	Literature Test Set for Sociological Literature

Search	Strategy	Records
1	su(autonomy OR privacy OR sharing OR disclosure OR identity OR confidentiality) AND su((perception OR attitude OR opinion OR fear OR preference OR concern)) AND su((genetic OR biobank OR DNA OR gene OR genome))	203

Search Strategy for ACM Digital Library

**Date**	6/30/2016
**Platform**	ACM Digital Library
**Collection**	The ACM Guide to Computing Literature:
**Notes**	

Search	Strategy	Records
1	(DNA gene genetic genome genomic) AND (private privacy confidential identity kin anonymous anonymity) AND (perception opinion poll survey perspective belief concern ethic fear)	333

Search Strategy for AnthroSource

**Date**	7/6/2016
**Platform**	Wiley Online Library / AnthroSource 2.0
**Collection**	Archive of all journals, newsletters, and bulletins of the American Anthropological Association.
**Notes**	Contacted webmaster@americananthro.org to download batch rather than single citation (7/6/16)<br>ti= Title; kw= Keyword

Search	Strategy	Records
1	(private OR privacy OR individual OR confidential OR autonomy OR personhood) AND (genetic OR DNA OR genome OR biobank) AND (perception OR opinion OR poll OR survey OR concern OR fear OR belief)	4,653
2	kw: (concern OR fear OR belief)	26
3	ti: (belief OR concern OR opinion OR perception OR perspective OR survey OR poll OR fear OR attitude)	1,626
4	kw: (private OR privacy OR individual OR confidential OR autonomy OR personhood OR self OR identity)	301
5	ti: (private OR privacy OR individual OR confidential OR autonomy OR personhood OR self OR identity)	1,506
6	kw: (genetics OR genetic OR HapMap OR DNA OR genome OR genomic OR genomics OR biobank OR biobanks OR bioethics)	45
7	ti: (genetics OR genetic OR HapMap OR DNA OR genome OR genomic OR genomics OR biobank OR biobanks)	181
8	kw: (concern OR fear OR belief) OR ti: (belief OR concern OR opinion OR perception OR perspective OR survey OR poll OR fear OR attitude) ***(2 OR 3)***	1,637
9	kw: (private OR privacy OR individual OR confidential OR autonomy OR personhood OR self OR identity) OR ti: (private OR privacy OR individual	1,705

Search	Strategy	Records
	OR confidential OR autonomy OR personhood OR self OR identity) ***(4 OR 5)***	
10	(kw: (concern OR fear OR belief) OR ti: (belief OR concern OR opinion OR perception OR perspective OR survey OR poll OR fear OR attitude)) OR (kw: (private OR privacy OR individual OR confidential OR autonomy OR personhood OR self OR identity) OR ti: (private OR privacy OR individual OR confidential OR autonomy OR personhood OR self OR identity)) ***(8 OR 9)***	3,300
11	(ethnography OR ethnology OR ethnologist OR bioethnology)	34,845
12	(kw: (concern OR fear OR belief) OR ti: (belief OR concern OR opinion OR perception OR perspective OR survey OR poll OR fear OR attitude)) OR (kw: (private OR privacy OR individual OR confidential OR autonomy OR personhood OR self OR identity) OR ti: (private OR privacy OR individual OR confidential OR autonomy OR personhood OR self OR identity)) OR (ethnography OR ethnology OR ethnologist OR bioethnology) ***(10 OR 11)***	36,382
13	((kw: (concern OR fear OR belief) OR ti: (belief OR concern OR opinion OR perception OR perspective OR survey OR poll OR fear OR attitude)) OR (kw: (private OR privacy OR individual OR confidential OR autonomy OR personhood OR self OR identity) OR ti: (private OR privacy OR individual OR confidential OR autonomy OR personhood OR self OR identity)) OR (ethnography OR ethnology OR ethnologist OR bioethnology)) AND (ti: (genetics OR genetic OR HapMap OR DNA OR genome OR genomic OR genomics OR biobank OR biobanks)) ***(12 AND 7)***	56
14	((kw: (concern OR fear OR belief) OR ti: (belief OR concern OR opinion OR perception OR perspective OR survey OR poll OR fear OR attitude)) OR (kw: (private OR privacy OR individual OR confidential OR autonomy OR personhood OR self OR identity) OR ti: (private OR privacy OR individual OR confidential OR autonomy OR personhood OR self OR identity)) OR (ethnography OR ethnology OR ethnologist OR bioethnology)) AND (ti: (genetics OR genetic OR HapMap OR DNA OR genome OR genomic OR genomics OR biobank OR biobanks)) OR kw: (genetics OR genetic OR HapMap OR DNA OR genome OR genomic OR genomics OR biobank OR biobanks OR bioethics) ***(13 OR 6)***	92

Search Strategy: Communication & Mass Media Complete

**Date**	7/6/2016
**Platform**	EBSCOhost
**Database**	Communication & Mass Media Complete
**Notes**	SU= Subject Heading; KW= Author Supplied Keyword

Search	Strategy	Records
S1	SU (genetic OR DNA OR genome OR biobank OR genomic)	785

S2	SU (trust OR attitude OR opinion OR concern OR privacy OR individuality OR personhood OR biocommerce OR perception OR identity OR sentiment OR discourse OR survey OR constructionism)	43,986
S3	KW (genetic OR DNA OR genome OR biobank OR genomic)	280
S4	KW (trust OR attitude OR opinion OR concern OR privacy OR individuality OR personhood OR biocommerce OR perception OR identity OR sentiment OR discourse OR survey OR constructionism)	14,578
S5	S1 OR S3	892
S6	S2 OR S4	51,862
S7	S5 AND S6	137

Search Strategy: Modern Language Association

**Date**	7/6/2016
**Platform**	ProQuest
**Database**	MLA International Bibliography
**Notes**	

Search	Strategy	Records
S1	(genetic OR genome OR genomic OR DNA) AND (privacy OR private OR discourse OR opinion OR perception OR perspective)	104
S2	(genetic OR genome OR genomic OR DNA) AND (privacy OR private OR discourse OR opinion OR perception OR perspective OR attitude OR constructionism OR personhood)	106
S3	(genetic OR genome OR genomic OR DNA OR genetics) AND (privacy OR private OR discourse OR opinion OR perception OR perspective OR attitude OR constructionism OR personhood)	136
S4	(genetic OR genome OR genomic OR DNA OR genetics OR biobank) AND (privacy OR private OR discourse OR opinion OR perception OR perspective OR attitude OR constructionism OR personhood OR belief)	147
